# Disclosure of HIV status outcome of regular counseling in a cohort of patients attending HIV clinics

**DOI:** 10.1186/1742-4690-9-S1-P59

**Published:** 2012-05-25

**Authors:** Livingstone Ssali, Francis Wasagami, Agnes Kateeba, Sophie Nantume, Andrew Kiboneka

**Affiliations:** 1 The Aids Support Organisation, Taso, Kampala, Uganda; 2 Kampala International University Teaching Hospital, Kampala, Uganda

## Background

The African continent bears the greatest burden of HIV/AIDS in the world. Efforts by scientists to find a vaccine for curing the pandemic have proved futile to date. The prevalence in Uganda stands at 6.4%in Adults and 0.7 % in Children, and about 1.1 million Ugandans are living with HIV/AIDS. The AIDS Support Organization started in 1987. Non disclosure of HIV sero status affects uptake of HIV/AIDS health services, increases stigma and discrimination.

## Methodology

A retrospective cohort study was done to review records of patients newly registered between January and December 2007 from Management Information System. We analyzed records of patients who had not previously disclosed their HIV/AIDS Sero status at the time of entry into TASO HIV/AIDS clinic. These patients routinely received counseling services for a period of 36 months to assess their disclosure status.

## Results

Out of 1413 patients counseled, 117(8.3%) patients had not disclosed their HIV sero status, 27(23%) patients were sexually active. During the first individual counselling sessions, patients were given information on condom use 19%, septrine prophylaxis 18%, sexually transmitted infections 15%, family planning 12%, and antiretroviral therapy 9%, safe water 8%, abstinence 5%, life skills 4%, prevention of mother to child transmission 4%, Tuberculosis 4%, voluntary counselling and testing 2%, and faithfulness 1%. Significant number of these sessions focused on opportunistic infections 39%, disclosure 15%, antiretroviral therapy 14%, drug therapy 9%, STD and HIV prevention 5%, nutrition 4% and welfare 4%, discordance 2%, sexuality and abstinence 3%. Other indirect interventions included HIV prevention sensitization through formation of peer support groups, drama sensitizations, group counseling and health talks during clinics. After 36 months of follow-up, 65-56% of 117 patients had disclosed their HIV sero status. Disclosure of HIV status is statistically associated with the number of counseling sessions (p=0.008). Average number of counseling sessions was 6 sessions. Patients who had not disclose after 36months recorded an average of 3 counselling sessions. Revealing of HIV sero status is statistically associated by sex, more female reveal their HIV Sero status in as short life span compared to males (p=0.002).

## Conclusions

The number of counseling sessions someone receives is associated with supported disclosure. Female patients reveal their HIV sero status in a shorter time span compared to males. Integration and more frequent provision of counseling services to patients in HIV/AIDS care and treatment enables them to make informed decisions regarding disclosure of their HIV sero status to their family members, sexual partners, friends and others. This has created support systems to clients and therefore reducing further spread of HIV.

